# A Hybrid Deep Learning Approach to Identify Preventable Childhood Hearing Loss

**DOI:** 10.1097/AUD.0000000000001380

**Published:** 2023-06-15

**Authors:** Felix Q. Jin, Ouwen Huang, Samantha Kleindienst Robler, Sarah Morton, Alyssa Platt, Joseph R. Egger, Susan D. Emmett, Mark L. Palmeri

**Affiliations:** 1 Department of Biomedical Engineering, Duke University, Durham, North Carolina, USA; 2 Department of Audiology, Norton Sound Health Corporation, Nome, Alaska, USA; 3 Department of Otolaryngology—Head and Neck Surgery, University of Arkansas for Medical Sciences, Little Rock, Arkansas, USA; 4 Duke Global Health Institute, Durham, North Carolina, USA; 5 Department of Biostatistics and Bioinformatics, Duke University School of Medicine, Durham, North Carolina, USA; 6 Department of Head and Neck Surgery and Communication Sciences, Duke University School of Medicine, Durham, North Carolina, USA; 7 These Authors contributed equally to this work.

**Keywords:** Children, Deep learning, Hearing, Layperson guided, Screening, Tympanometry

## Abstract

**Objective:**

Childhood hearing loss has well-known, lifelong consequences. Infection-related hearing loss disproportionately affects underserved communities yet can be prevented with early identification and treatment. This study evaluates the utility of machine learning in automating tympanogram classifications of the middle ear to facilitate layperson-guided tympanometry in resource-constrained communities.

**Design:**

Diagnostic performance of a hybrid deep learning model for classifying narrow-band tympanometry tracings was evaluated. Using 10-fold cross-validation, a machine learning model was trained and evaluated on 4810 pairs of tympanometry tracings acquired by an audiologist and layperson. The model was trained to classify tracings into types A (normal), B (effusion or perforation), and C (retraction), with the audiologist interpretation serving as reference standard. Tympanometry data were collected from 1635 children from October 10, 2017, to March 28, 2019, from two previous cluster-randomized hearing screening trials (NCT03309553, NCT03662256). Participants were school-aged children from an underserved population in rural Alaska with a high prevalence of infection-related hearing loss. Two-level classification performance statistics were calculated by treating type A as pass and types B and C as refer.

**Results:**

For layperson-acquired data, the machine-learning model achieved a sensitivity of 95.2% (93.3, 97.1), specificity of 92.3% (91.5, 93.1), and area under curve of 0.968 (0.955, 0.978). The model’s sensitivity was greater than that of the tympanometer’s built-in classifier [79.2% (75.5, 82.8)] and a decision tree based on clinically recommended normative values [56.9% (52.4, 61.3)]. For audiologist-acquired data, the model achieved a higher AUC of 0.987 (0.980, 0.993), had an equivalent sensitivity of 95.2 (93.3, 97.1), and a higher specificity of 97.7 (97.3, 98.2).

**Conclusions:**

Machine learning can detect middle ear disease with comparable performance to an audiologist using tympanograms acquired either by an audiologist or a layperson. Automated classification enables the use of layperson-guided tympanometry in hearing screening programs in rural and underserved communities, where early detection of treatable pathology in children is crucial to prevent the lifelong adverse effects of childhood hearing loss.

## INTRODUCTION

Hearing loss affects over 1.5 billion people worldwide, and the burden of disease falls disproportionately on individuals living in low- and middle-income countries (GBD 2019 Hearing Loss Collaborators 2021). Hearing loss in children has lifelong consequences, including delayed cognitive development, delayed speech and language, reduced academic performance, increased likelihood of unemployment, and higher rates of social isolation and depression compared to peers (Järvelin et al. 1997; Furlonger 1998; National Research Council et al. 2004; Theunissen et al. 2011; Cardon et al. 2012; Emmett & Francis 2015; Linszen et al. 2016; Vas 2017; Idstad & Engdahl 2019; Blazer 2020; Glick & Sharma 2020; Yong et al. 2020a).

The WHO estimates that 60% of hearing loss in children is preventable, with infection-related hearing loss accounting for 31% of all hearing loss in children (Sensory Functions, Disability, & Rehabilitation 2021). For this reason, school hearing screening is essential for early identification and treatment (Yong et al. 2020b). However, screenings are only mandated in a few regions globally, and screening protocols are not standardized, with current screening methods often falling short at identifying children with preventable, infection-related hearing loss (Yong et al. 2020a).

Tympanometry is an audiometric test that uses an acoustic stimulus and variations in air pressure to evaluate the middle ear. It is an objective measure for identifying middle ear conditions associated with infection-related hearing loss, such as fluid behind the tympanic membrane (eardrum), Eustachian tube dysfunction (retraction), or perforation (hole) in the eardrum. To perform tympanometry, a probe with a silicone tip is inserted into the ear canal to obtain an airtight seal. Sound pressure is systematically applied to evaluate the compliance of the tympanic membrane and detect middle ear pathology. The acquired compliance curves (tracings) can be classified into types A, B, and C (Jerger 1970). Classifications of types B and C indicate pathology (e.g., fluid or retraction, respectively), and need clinical follow-up. Tympanometry is traditionally performed only by trained audiologists in clinical settings and is not typically part of screening programs due to lack of screening guidelines, cost, complexity, and the lack of decision-making support for layperson use.

In our recently completed cluster-randomized trials of hearing screening and referral methods in school-aged and preschool children in rural Alaska, tympanometry was included in the screening protocol because the population in this trial experiences a disproportionately high burden of infection-related hearing loss, analogous to many rural, resource-constrained settings globally (Emmett et al. 2019, 2022). From these trials, we determined that the inclusion of tympanometry with pure-tone screening significantly increased the accuracy of school hearing screening when compared to gold standard audiometric evaluation (Robler et al. 2023). Enabling laypersons to collect diagnostic quality tympanometry data via an automated guidance and tympanometry classification interpretation system could significantly improve hearing screening accessibility in low-resource communities globally, where audiologist capacity is limited.

During these recent trials, we collected a dataset of nearly 10,000 tympanometry tracings acquired by the audiologist and layperson from 1635 children. Using this dataset, we developed and trained an interpretable, hybrid deep learning model to automatically classify tympanometry tracings. Our goal was to use machine learning methods to support interpretation of layperson tympanometry data with sensitivity comparable to a trained audiologist to ultimately improve hearing screening in underserved areas.

## MATERIALS AND METHODS

### Data Acquisition

Tympanometry data were collected from 1635 participants, aged 3 to 21 years, as part of two cluster-randomized school hearing screening trials in the Norton Sound region of rural Alaska with K-12 and preschool children (NCT03309553, NCT03662256) (Emmett et al. 2019, 2022). Both trials were approved by Institutional Review Boards. All children (preschool–12) attending preschool or grade school in Bering Strait School District with parental informed consent and child assent were eligible. For children K-12th grade, the trial spanned two academic years. The trial for preschool children was added in the second year of the K-12 trial. Hearing screening occurred annually, and thus, children enrolled in the first year of the trial could have participated in the study over 2 years if present in school both years.

On screening day, participation in the study involved completion of two screening protocols (the school hearing screening and a mobile health screen with tympanometry), as well as a gold standard audiometric assessment that included otoscopy, tympanometry, and audiometry performed by a trained audiologist. Both a trained audiologist and a layperson independently acquired tympanometry tracings for each child to understand performance of target layperson-acquired screening results against trained audiologist assessment. With both layperson- and audiologist-acquired tympanograms, as well as potential participation over two trial years, tympanometry could have been collected up to four times for the same child. Both the audiologists and lay screeners were blind to the others’ results. Tympanometry was collected using a commercial tympanometer (Otometrics Otoflex 100; Natus; Taastrup, Denmark).

Both layperson- and audiologist-acquired tympanograms were interpreted and classified into type A, B, or C, using prescribed normative values (FitzZaland & Zink 1984), with the latter two generating a follow-up referral. Laypersons received training and instruction before data collection and were given limited support throughout collection. Tracing and classification data were aggregated and anonymized. The audiologist’s interpretation of audiologist-acquired tracings was used as the gold standard.

### Data Preprocessing

**Fig. 1.**
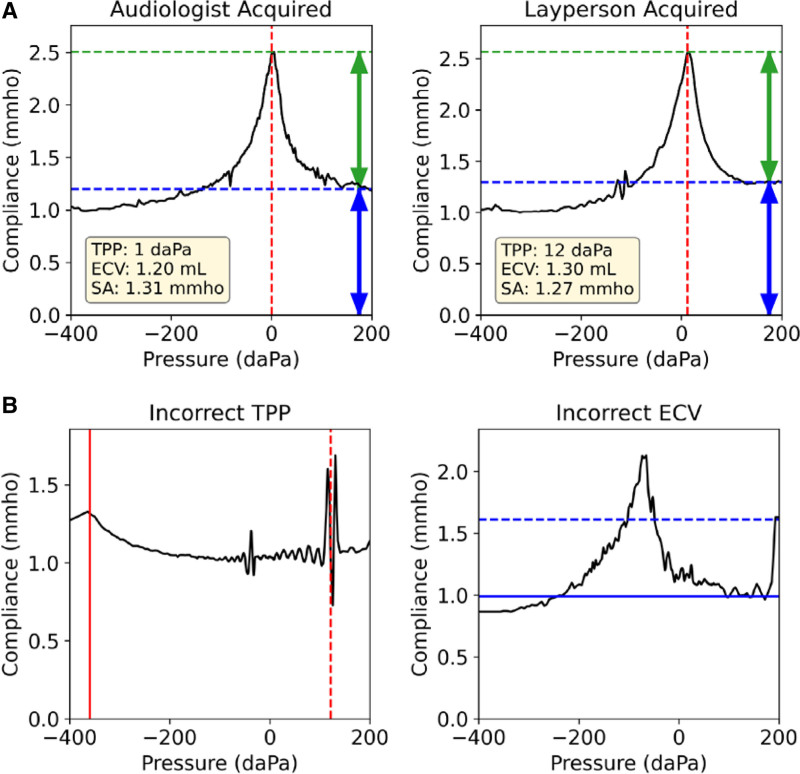
Tympanometry tracing with labeled attributes. A, Two tracings acquired from the same ear by a trained audiologist (left) and a layperson (right). Tympanometric peak pressure (TPP) is shown in red, equivalent ear canal volume (ECV) in blue, and static admittance (SA) in green. Both tracings are of Type A classification, i.e. pass on screening. Note: Tympanogram compliance values are displayed as uncompensated, where the ECV (blue section) is not subtracted out. B, Two representative noisy tracings with incorrect TPP and ECV calculations by commercial software, leading to an incorrect automated commercial software diagnosis. Commercial software values are shown as dotted lines, manually identified values are shown as solid lines. An incorrect TPP is calculated due to an artifactual spike near 120 daPa (left). An incorrect ECV is calculated near 1.6 mmho due to an artifactual spike at +200 daPa (right).

Commercial software associated with the tympanometer (Otosuite; Natus) analyzed each tracing and provided the following tympanometry attributes: tympanometric peak pressure (TPP), tympanometric width, static admittance (SA), and equivalent ear canal volume (ECV). ECV, in milliliters, is converted from the value of the uncompensated compliance, in mmho, at +200 daPa. The software also predicted tympanometric type classification. Figure 1 shows an example tracing with labeled tympanometry attributes. The TPP and ECV were manually identified for all tracings. The software also provides a TPP and ECV interpretation; however, these values were not always reliable due to signal noise (Fig. 1B).

The tympanometer used a variable sampling rate, higher around a detected peak, so all tracings were resampled to a grid of 600 uniformly spaced pressure values from –399 to 200 daPa. The resampled tracings were low pass filtered using a fourth-order Butterworth filter with critical frequency 1/25 daPa^–1^ to reduce high-frequency noise.

### Hybrid Deep Learning Model

Deep learning has shown impressive accuracy and performance in 1D and 2D signal detection problems such as audio/image classification (LeCun et al. 2015). However, models have poor interpretability and are commonly described as a “black-box.” In contrast, decision trees are inherently transparent and interpretable, but they have difficulty processing raw data such as audio amplitude tracings or image pixel intensity values. Our hybrid approach incorporates the flexibility of deep learning with the interpretability of decision trees. In tympanometry, attributes such as TPP and ECV are highly predictive of A, B, or C tympanometry readings. We designed a deep learning model that takes a tympanogram as input and predicts the TPP and ECV.

To capture predictive uncertainty, we adopted the approach used by Kendall and Gal (2017). For each attribute, the deep learning model outputs a second quantity estimating the uncertainty. The loss function to train such a model is


$$\begin{array}{l} L=\;\frac{1}{N}\sum_{i=1}^{N}\left[\frac{{\left(y_{i}-{\hat{y}}_{i}\right)}^{2}}{{\hat{\sigma }}_{i}^{2}}+\log{\hat{\sigma }}_{i}^{2}\right], \end{array}$$

where *ŷ* is the target value, and $\hat{\sigma }$ is the predicted value, and is the predicted uncertainty. A large uncertainty decreases the penalty for incorrect predictions, and a regularization term prevents the network from simply maximizing the predicted uncertainty. The predictive uncertainty *σ* represents the standard deviation of a Gaussian probability distribution (Kendall & Gal 2017). Our deep neural network architecture is based on the widely used ResNet (He et al. 2016), but with 1D rather than 2D layer operations. A detailed description of the neural network architecture and training is described (see Methods and Table 1 in Supplemental Digital Content, http://links.lww.com/EANDH/B143).

Predicted TPP and ECV values along with their uncertainties were passed into a decision tree model to predict the tympanometry type. Each decision tree was limited to a depth of three to maintain interpretability and generalizability. To adjust the sensitivity-specificity tradeoff, we used a variable weighting for type A versus type B/C.

### Clinically Normative Values

Clinically normative values (SA < 0.2 mmho, TPP ≤ –200 daPa, and ECV > 2.0 mL) (FitzZaland & Zink 1984) were used as part of the screening process in the trials to distinguish between abnormal and normal tracings. For comparison purposes, we created a simple decision tree that assigns A/B/C type following these clinically normative thresholds.

### Implementation

Data processing was performed in Python 3.8 using the NumPy, SciPy, and Pandas packages. The deep learning model was designed and trained using the PyTorch package with NVIDIA V100 GPU hardware. The decision tree model was constructed and analyzed using the Scikit-learn package.

### Evaluation of Diagnostic Accuracy

Performance metrics of the machine learning classifier were assessed using ten-fold cross-validation. Assignment to the ten folds was randomly sampled at the level of the child to maintain independence of test and training sets.

Percent correctly classified (accuracy) was calculated as both three-level (types A, B, C) and two-level (pass/refer) comparisons. Sensitivity, specificity, and predictive values were calculated by treating type A as pass and types B and C as refer. Metrics were calculated for the tympanometry software’s interpretation, the simple decision tree based on normative values, and the machine learning model. An ROC curve was generated for the machine learning model by varying the A versus B/C class weighting, and an area under the curve (AUC) after combining splits was calculated (Figure 1 in Supplemental Digital Content 1, http://links.lww.com/EANDH/B143); the reported sensitivity, specificity, and predictive values are at the point that maximizes the Youden’s index (sum of sensitivity and specificity). We report predictive values and Youden’s index because this is a skewed dataset (90.0% were type A, 7.2% were type B, and 2.8% were type C); these metrics better capture performance beyond accuracy. AUC was not calculated for the simple decision tree or the built-in software because they do not have adjustable classification thresholds.

Generalized estimation equations with binomial distribution and identity link (with independence working correlation) were specified to calculate the proportion correctly classified, sensitivity, specificity, and predictive values with confidence intervals to account for repeated measurements within child (ear and year). Confidence intervals for AUC were generated using (child-level) cluster bootstrapping with 500 samples (Carpenter & Bithell 2000). All performance summaries were computed using Stata 17 software.

## RESULTS

**Fig. 2.**
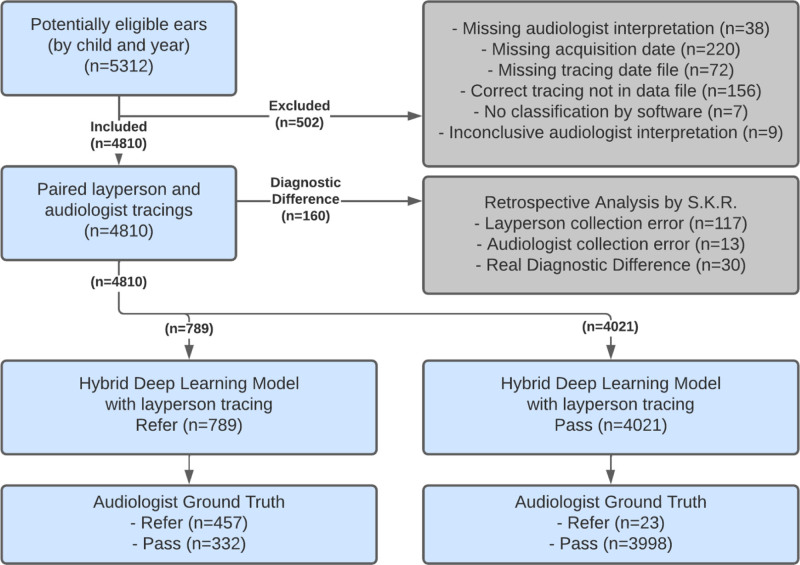
Standards for Reporting of Diagnostic Accuracy Studies diagram.

The distribution and exclusion steps applied to the dataset are outlined in STARD diagram (Fig. 2). The complete dataset from 1635 children examined a total of 5312 ears by both audiologist and layperson for a total of 10,624 tympanometer tracings. Four hundred forty-eight ears were excluded due to missing tracing date files, missing tracings, and missing acquisition dates. Fifty-four ears were excluded due to missing audiologist interpretation, missing software classification, and inconclusive audiologist interpretation In total, these 502 exclusions yielded 4810 pairs of audiologist and layperson tracings (9620 total tracings) from 1576 children. Of the 4810 ears analyzed, 4330 (90.0%) were type A, 345 (7.2%) were type B, and 135 (2.8%) were type C, as determined by the audiologist interpretation of the audiologist’s tracing (see Table 2 in Supplemental Digital Content 1, http://links.lww.com/EANDH/B143, for sociodemographic and clinical characteristics of the study sample). The model trained on these data have been made available to the public for reproducibility purposes (see Methods in Supplemental Digital Content 1, http://links.lww.com/EANDH/B143).

### Deep Neural Network Estimation of TPP and ECV

**Fig. 3.**
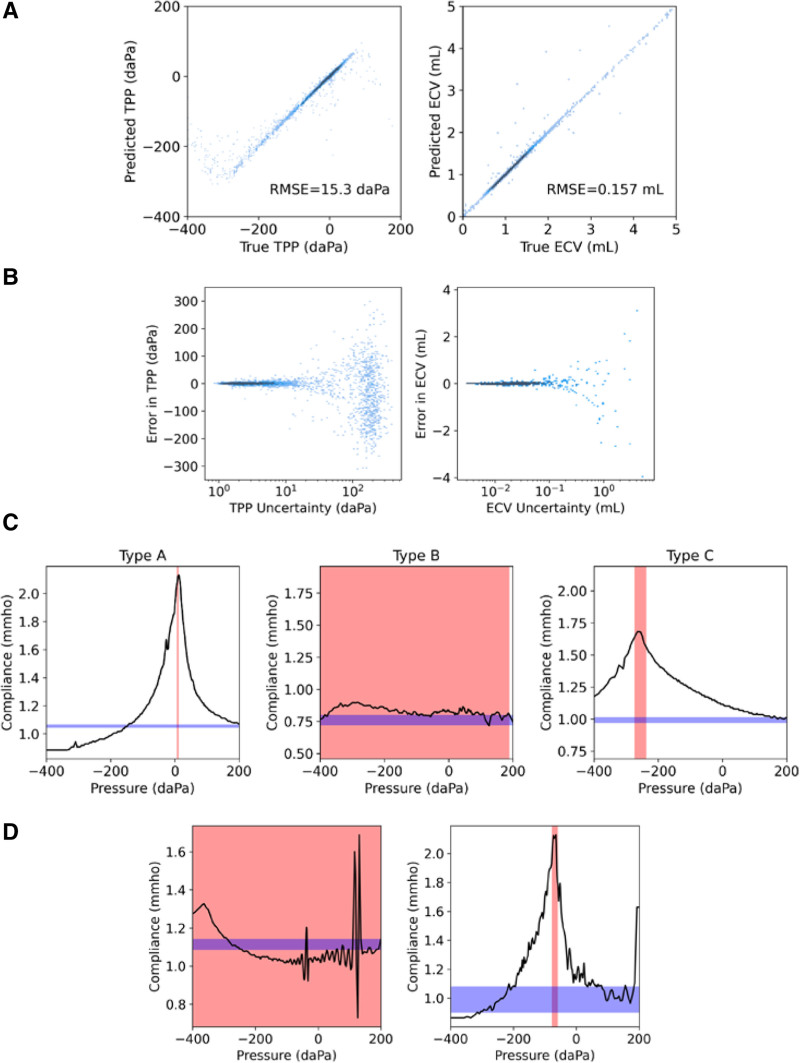
Output of the deep neural network. A, Predicted tympanometric peak pressure (TPP) and ear canal volume (ECV) values versus ground truth values. Agreement between predicted and true values is indicated by points lying along the equivalence line and by low root-mean-square-error (RMSE). B, Predicted TPP and ECV uncertainty values versus error. C, Predicted TPP (red) and ECV (blue) and their respective uncertainties, for example, tracings of each classification type. The width of each colored region corresponds to twice the predicted uncertainty (standard deviation). The Type B tracing had a TPP uncertainty >300 daPa and thus, uncertainty highlights nearly the full displayed range. D: Examples of representative noisy data with uncertainty highlighting, same as (C). Note from Figure 1 that the commercial FDA cleared software incorrectly assigns a TPP near 120 daPa due to artifact (left) and incorrectly assigns the ECV value to 1.6 mmho due to artifact (right).

For each tympanometer tracing, estimated TPP and ECV values by the trained deep neural network were plotted against the target ground truth values in Figure 3A. Most points fell along the equivalence line, indicating good agreement between predicted and true values. The root-mean-square-error between predicted and target values was 15.3 daPa for TPP and 0.157 mL for ECV. Ears with significant retraction had large negative TPP values, and the peak was harder to detect with a slope only present on one side. In Figure 3B, error is plotted against the deep learning predicted uncertainty metric. This plot demonstrates that higher predicted uncertainty was correlated with higher error.

Examples of types A, B, and C tracings are shown in Figure 3C, with the predicted TPP and ECV values and uncertainties shown in red and blue, respectively. For type B tracings, the network output a TPP uncertainty >300 daPa, covering the entire pressure sweep range and indicating the lack of a well-defined peak. Figure 3D shows the effect of uncertainty on noisy data. The commercial software incorrectly used the artifactual value at +200 daPa as ECV, whereas our model provided an uncertainty range within the expected ECV range. Due to noise, the commercial software incorrectly assigned the TPP value, whereas our model output a high uncertainty which signified that a peak did not exist.

### Decision Tree Classifiers

**Fig. 4.**
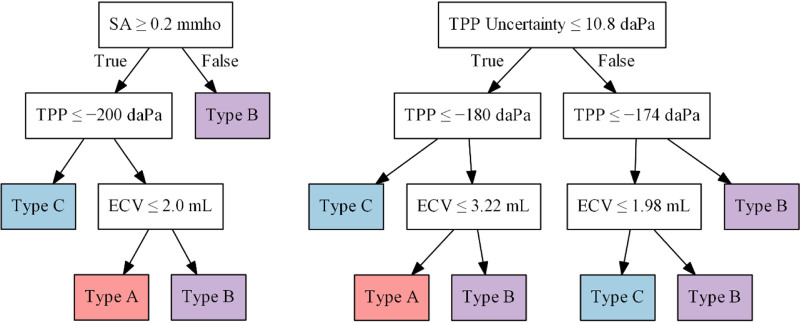
Simple and trained decision trees. Left, A simple decision tree based on clinically recommended normative values. Right, The trained decision tree of the hybrid deep learning model. Uncertainty is a feature output by our deep learning model which can be plugged into our decision tree similar to other numerical features (TPP uncertainty).

Figure 4 shows the simple decision tree based on normative values compared to an example decision tree trained on the deep learning features. Uncertainty is a feature output by our deep learning model which can be plugged into our decision tree similar to other numerical features. We observed that the trained decision tree shared a similar structure with the simple tree: both split at the first level by a measure of peakedness (SA or TPP uncertainty), by TPP at the second level, and by ECV at the third level. We noted that the decision tree model always preferred to split using TPP uncertainty instead of SA if both values were provided.

Table 1 shows the classification performance metrics of the trained hybrid decision tree and deep learning model when applied to audiologist- and layperson-acquired tracings. The performance of the built-in tympanometer software and the simple decision tree are also shown for comparison. The sensitivities of the two comparison methods were <80% using audiologist- or layperson-acquired tracings. The machine learning model achieved 95.2% (93.3, 97.1) sensitivity for both the audiologist and layperson-acquired tracings. The AUC of the machine learning classifier was 0.987 (0.980, 0.993) using audiologist tracings and 0.968 (0.955, 0.978) using layperson tracings [see Table 3 in Supplemental Digital Content 1, http://links.lww.com/EANDH/B143, for comparisons of subject characteristics between each fold of cross-validation; see Tables 4–7 in Supplemental Digital Content 1, http://links.lww.com/EANDH/B143, for confusion matrices tested for each assignment rule (simple decision tree, built-in software, hybrid deep learning) and each data source (layperson- vs. audiologist-acquired)]. The three-level class-specific sensitivities and specificities are also provided in Tables 4 and 5 in Supplemental Digital Content 1, http://links.lww.com/EANDH/B143. For the hybrid deep learning model, all class-specific sensitivities and specificities were >90%, with the exception of sensitivity for type C tracings for both layperson and audiologist data. However, these values were superior to respective values for the simple decision tree and built-in software. As noted previously, Figure 3A shows that peaks with large negative TPP, that is, type C tracings, were harder to detect accurately.

**TABLE 1.** Classification performance with audiologist interpretation and audiologist acquisition as gold standard*

	Audiologist’s Acquisition			Layperson’s Acquisition		
Automated Clinical Decision Support Tool	Built-in Software†	Simple Decision Tree‡	Our Hybrid Deep Learning	Built-in Software†	Simple Decision Tree‡	Our Hybrid Deep Learning
Accuracy (A/B/C)	93.5 (92.8, 94.2)	92.5 (91.8, 93.2)	97.0 (96.5, 97.4)	92.2 (91.5, 93.0)	91.5 (90.7, 92.2)	91.7 (90.9, 92.5)
Accuracy (pass/refer)	94.2 (93.5, 94.9)	92.8 (92.0, 93.5)	97.5 (97.0, 97.9)	93.1 (92.3, 93.8)	91.8 (91.0, 92.6)	92.6 (91.9, 93.4)
Sensitivity	77.7 (74.0, 81.4)	63.3 (59.0, 67.6)	95.2 (93.3, 97.1)	79.2 (75.5, 82.8)	56.9 (52.4, 61.3)	95.2 (93.3, 97.1)
Specificity	96.0 (95.4, 96.6)	96.0 (95.4, 96.6)	97.7 (97.3, 98.2)	94.6 (93.9, 95.3)	95.7 (95.1, 96.3)	92.3 (91.5, 93.1)
Youden’s Index	73.7 (70.0, 77.4)	59.3 (54.9, 63.7)	92.9 (90.9, 94.9)	73.8 (70.1, 77.5)	52.6 (48.1, 57.1)	87.5 (85.4, 89.6)
Positive Predictive Value	68.4 (64.5, 72.3)	63.9 (59.5, 68.2)	82.3 (79.2, 85.5)	61.9 (58.0, 65.7)	59.2 (54.7, 63.7)	57.9 (54.5, 61.4)
Negative Predictive Value	97.5 (97.0, 98.0)	95.9 (95.4, 96.5)	99.5 (99.2, 99.7)	97.6 (97.2, 98.1)	95.2 (94.6, 95.9)	99.4 (99.2, 99.7)
AUC§			0.987 (0.980, 0.993)			0.968 (0.955, 0.978)

* Population is based on the full N = 4810 tympanometry ear tracing dataset collected over 2 years from 1576 children in the Bering Strait School District of Rural Alaska (Emmett et al. 2019, 2022). Audiologist gold standard determined 4330 pass (negative) and 480 (positive) referrals.

† Built-In Software refers to the proprietary built-in decision support software from Otosuite, (Natus; Taastrup, Denmark) software provided with their FDA cleared tympanometer system.

‡ Simple decision tree refers to clinically normative values: Static Admittance (SA) < 0.2 mmho, Tympanometric peak pressure ≤ –200 daPa, and ear canal volume > 2.0 mL

§ AUC can only be reported if adjustable decision thresholds exist.

AUC, area under curve; ECV, ear canal volume; TPP, tympanometric peak.

published online ahead of print June 15, 2023.

### Diagnostic Differences Between Layperson- and Audiologist-acquired Tracings

We retrospectively analyzed the full 4810 audiologist-layperson paired tympanometry tracings to look for diagnostic differences. A significant diagnostic difference was defined as a difference in the tracing itself that would have led to a different tympanometry classification by an audiologist. In total, 160 cases (3.3%) were identified with significant diagnostic differences between the layperson-acquired tracing compared to the matching tracing acquired by the audiologist from the same child and ear. Of the 160 tracing pairs, 73% (117 cases) were due to layperson error, with most attributed to incorrectly pressing the probe against the ear canal wall during measurement. There were audiologist errors in 8% (13 cases) of the identified cases, and 19% (30 cases) had presumably real diagnostic differences, which are inherent to tympanometric measurements (e.g., variations in negative pressure between measurement times). Findings of pathology (both presence of middle ear disease and tympanograms classified as type B/C by audiologist interpretation) were more frequent for child/ears where diagnostic differences were found (see Table 8 in Supplemental Digital Content 1, http://links.lww.com/EANDH/B143).

## DISCUSSION

This is the first work to demonstrate that automated clinical decision support can make it possible for laypersons to perform screening tympanometry in rural underserved populations with sensitivity similar to a trained audiologist. The inclusion of tympanometry in childhood hearing screenings may help identify and refer children with preventable, infection-related hearing loss, which if left untreated could lead to lifelong consequences. We leveraged the largest dataset of tympanometry tracings reported in the literature to develop and train a hybrid deep learning model to classify tympanograms into the type A/B/C categories and assign the appropriate referral status.

Recently, it was demonstrated that tympanometry could be performed using a smartphone-based platform (Chan et al. 2022), an important step towards layperson-based screening. To see whether our automated algorithm was compatible with data collected by Chan et al.’s device, we applied the model to the openly shared data provided in their article (see Results in Supplemental Digital Content 1, http://links.lww.com/EANDH/B143). We found that our model was flexible and robust, accurately classifying the provided commercial and smartphone tympanometer data.

We estimate the hypothetical clinical impact of our algorithm on our study population using the metrics in Table 1. If laypersons used our machine learning model instead of the commercial tympanometer’s software, 77 additional ears with preventable hearing loss would have been identified at a cost of 98 additional false positive ears to be assessed by further triage. The pros of preventing potential childhood hearing loss in 77 ears outweigh the cost of triaging an additional 98 false positives. Additionally, we investigate the projected economic impact of our work in decreasing the cost of tympanometry screening. Our model is open source under Apache 2.0 and can be applied to other cheaper devices. For instance, we apply our model to a smartphone-based tympanometer whose material costs $28. Comparatively, commercial tympanometers can cost $2000–$5000 (Chan et al. 2022). We show that laypersons can triage normal and abnormal tympanometry readings which are traditionally performed by audiologists. These cost savings are realizable for both low-resource areas as well as high-income settings to reduce the burden on highly trained professionals.

Otoscopy is an alternative tool for detecting ear disease that uses an optical lens to examine the tympanic membrane and middle ear. Unlike tympanometry, otoscopy is difficult to implement in screening programs because of the significant training required for proficiency. Even with significant training, otoscopy can be challenging to perform and interpret. In one study, fellowship-trained neurotologists were only correct 72% of the time in identifying normal eardrums (Moberly et al. 2018). In contrast, we have shown that tympanometry tracings can be acquired by a layperson and objectively interpreted by a machine learning algorithm for screening purposes with statistically equivalent sensitivity to audiologist-acquired tracings and near-audiologist level AUC values. Specificities of all algorithms were high, >0.90, which is important for screening programs.

In contrast to the large body of work applying machine learning methods to analyze otoscopic images, there are relatively few studies that explore automatic classification of tympanograms. One study trained a random forest classifier to predict clinician findings from raw tympanometry attributes and combined these predictions with another classifier for digital otoscopy (Binol et al. 2020). This study was limited by a low sample size (N = 73). The authors reported an accuracy of 76.7% for tympanometry classification. Another recent study tested various machine learning techniques for detecting otitis media with effusion from wideband tympanometry data, with a dataset size of 672 ears (Grais et al. 2021). The authors reported accuracies between 74% and 82%, with neural networks performing slightly better than random forest or k-nearest neighbors methods. By comparison, our study used a large tympanometry dataset (n = 9620 tracings) with multiple pathologies and found an accuracy of 97.5% (97.0, 97.9) and 92.6% (91.9, 93.4) for audiologist and layperson tracings, respectively. The machine learning model’s sensitivity was statistically equivalent between audiologist-acquired and layperson-acquired tracings, suggesting that layperson-guided tympanometry screening is valid using a fully automated tympanogram classifier for clinical decision support.

Tympanometry is a technology currently limited to high resource environments as a tool used primarily by trained audiologists in the clinical setting. We envisioned the utilization of tympanometry in rural low-resource settings and considered two primary challenges: (1) increased noise and artifacts associated with layperson operation, and (2) the necessity for automated referral decision-making. A key disadvantage of machine learning classifiers such as random forest, support vector machine, and end-to-end deep neural network is their lack of interpretability. Therefore, we developed a hybrid model that paired a transparent decision tree with interpretable and clinically relevant features extracted by deep learning. Deep learning was selected over a manually engineered algorithm for feature extraction to leverage uncertainty estimation, greater robustness, and flexibility for retraining/fine-tuning. The optimized decision tree had a similar branching pattern to clinically normative guidelines. We believe the flexibility of the hybrid deep learning approach may allow it to perform well on noisy data obtained from layperson screeners regardless of the tympanometric device used.

Laypeople were not expected to acquire perfect tracings for every patient, especially without the rigorous clinical training and experience of an audiologist. To assess the limitations of layperson-acquired tracings, we screened out tracings with a significant diagnostic difference between layperson and audiologist tracings. The observed 3.1% error rate may be considered an upper bound on the accuracy of any classifier on this dataset. The sources of diagnostic differences between tracings include probe position or participant motion during acquisition. In future work, tracing quality checks should be implemented on tympanometer software or hardware to mitigate these errors and guide laypersons for improved acquisition of tympanometry results.

Another limitation is that this tympanometry dataset was collected using only one set of diagnostic commercial hardware and software on one rural population. Future work should be completed using more diverse tympanometers and populations because we expect the need for additional training of the automatic classification with lower cost tympanometers, those run with alternative parameter settings (e.g., pressure sweep, pump speed), and in populations that have differing presentations of middle ear disease. Neural networks and regression trees are both statistical methods, and performance can be degraded by significant shifts in disease presentation as well as population prevalence. Finally, variances around the performance metrics for classification accuracy may be underestimated due to the overlap in training data used for the 10-fold cross-validation process; therefore, 95% confidence intervals should be interpreted with caution.

## CONCLUSIONS

Infection-related hearing loss in children is preventable if detected and treated early. We demonstrate the laypersons’ ability to capture diagnostic quality tympanometry data and the utility of using a hybrid deep learning method for automated classification. Our results suggest that hybrid deep learning could facilitate the integration of accurate, layperson-guided tympanometry into hearing screening programs around the world, especially for low-resource and rural communities.

## ACKNOWLEDGMENTS

The study was funded by the Patient-Centered Outcomes Research Institute (PCORI AD-1602-34571) and by the Duke Global Health Institute AI Pilot Research Grant.

## Supplementary Material


