# Supplementary material for: A Hybrid Deep Learning Approach to Identify Preventable Childhood Hearing Loss
Source: Ear Hear. 2023 Jun 15;44(5):1262–70. doi: 10.1097/AUD.0000000000001380 (PMC10426782; doi:10.1097/AUD.0000000000001380)

Supplemental Digital Content

Methods: Deep Neural Network Architecture and Training
Results: Model Application to Smartphone Tympanometer Device Data
Table 1. Deep Neural Network Architecture
Table 2. Sociodemographic and clinical characteristics of study sample at the child and child/year level
Table 3. Comparison of demographic and clinical characteristics across cross-validation folds (ear level)
Table 4. Confusion matrix for 3-level classification using layman tracings
Table 5. Confusion matrix for 3-level classification using audiologist tracings
Table 6. Confusion matrix for pass/refer classification using layman tracings
Table 7. Confusion matrix for pass/refer classification using audiologist tracings
Table 8. Comparison of sociodemographic and clinical characteristics between child/ear/years where tracings differed diagnostically between audiologist and layman (i.e. the 3.01%)
Figure 1. ROC curves of Hybrid Deep Learning Model

Methods: Deep Neural Network Architecture and Training

Our deep neural network takes the 600-point resampled tympanometry tracing as input, along with the low-pass filtered tracing and a vector of the sweep pressure in kPa at each point. The neural network architecture is detailed in eTable 1 and is roughly based on the widely-used ResNet but with 1D rather than 2D layer operations. Batch normalization and LeakyReLU were used after each convolution operation. Zero-padding of 1 was used only for the convolutions with kernel size 3. The network was trained using the Adam optimizer with learning rate 0.003 for 40 epochs and a batch size of 64. For tracings without an identifiable peak, a random value was sampled from the pressure sweep range at the beginning of each epoch. No additional data augmentation was used. The trained model is available at the following code repository:

https://gitlab.oit.duke.edu/mhealthtympanometer/mhealth-tymp-classifier

Results: Model Application to Smartphone Tympanometer Data

In work performed by (Chan, J., Najafi, A., Baker, M. et al. 2022), 50 pairs of tympanometer tracings were collected on a commercial device and on a smartphone-based hardware device. Expert audiologists classified the tympanometer tracing type to A, As, Ad, B, and C. Treating As and Ad tracing types as A, we apply our deep learning hybrid model approach to classify A, B, and C type. We achieve 100% agreement on commercial tracings. On the smartphone device tracings, our model only has one disagreement. Upon closer inspection, this tracing is significantly different from its paired commercial tracing. Review by S.K.R, an expert audiologist, suggests that this tracing is more consistent with Type B but could also be classified a shallow type A, consistent with higher uncertainty.

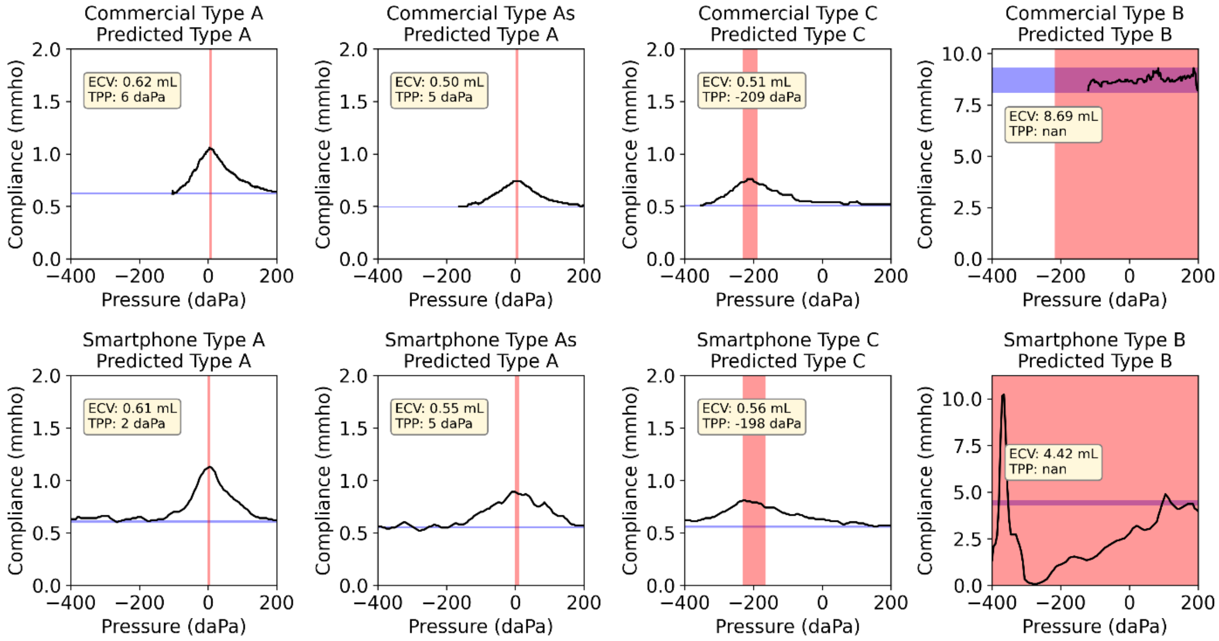

Representative commercial and smartphone tracings with deep learning model predictions

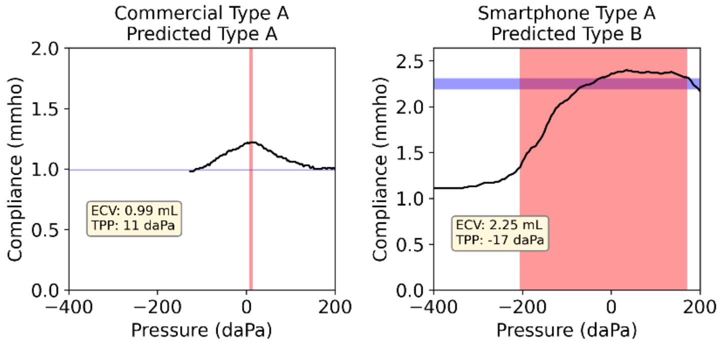

Single disagreement between model and ground truth defined by (Chan, J., Najafi, A., Baker, M. et al. 2022). Note that the smartphone tracing is significantly different from the commercial tracing. Review by S.K.R, an expert audiologist, shows the smartphone tracing has B type characteristics and further clinical investigation would be recommended.

Table 1. Deep Neural Network Architecture

Block	Layer	Output Channels	Kernel Size	Stride
Input Block	Conv1d	16	7	4
ResBlock 1	Conv1d	16	3	2
	Conv1d	16	3	1
	Add Conv1d	16	1	2
ResBlock 2	Conv1d	16	3	2
	Conv1d	16	3	1
	Add Conv1d	16	1	2
ResBlock 3	Conv1d	32	3	2
	Conv1d	32	3	1
	Add Conv1d	32	1	2
ResBlock 4	Conv1d	64	3	2
	Conv1d	64	3	1
	Add Conv1d	64	1	2
Output Block	Flatten	640		
	Linear	64		
	LeakyReLU			
	Linear	4		
Total parameters: 71604				

Table 2. Sociodemographic and clinical characteristics of study sample at the child and child/year level

Number of Children	**Total (*N*=1576)**
Grade category – N (%)	
ECE	140 (8.9%)
K-5	829 (52.6%)
6-8	317 (20.1%)
9-12	290 (18.4%)
Age category – N (%)	
3-6	409 (26.0%)
7-9	402 (25.6%)
10-12	339 (21.6%)
13-15	234 (14.9%)
16-18+	189 (12.0%)
Missing	3 (.%)
Sex	
Female, N(%)	750 (47.6%)
Male, N(%)	826 (52.4%)
Alaska Native/American Indian, N(%)	1510 (95.8%)
Highest education of any caregiver, N (%)	
<12 grade	91 (5.9%)
HS Diploma or GED	995 (64.6%)
Some College	306 (19.9%)
College Degree	148 (9.6%)
Missing	36 (.%)

**Number of Ears**	**Year 1 (N=2084)**	**Year 2 (N=2726)**	**Total (N=4810)**
Number of Child Observations	Year 1 (*N*=1086)[1]	Year 2 (*N*=1374)[1]	Total (*N*=2460)[2]
One ear	88 (8.1%)	22 (1.6%)	110 (4.5%)
Both ears	998 (91.9%)	1352 (98.4%)	2350 (95.5%)
Tympanometry-based referrals[3]			
Bilateral	64 (5.9%)	88 (6.4%)	152 (6.2%)
Unilateral	74 (6.8%)	102 (7.4%)	176 (7.2%)
No referral	948 (87.3%)	1184 (86.2%)	2132 (86.7%)
Middle ear disease (any ear), N(%)[3]	165 (15.2%)	228 (16.6%)	393 (16.0%)

[1]One observation per child
[2]Up to two observations per child
[3]Based on audiologist (ground truth) diagnostic assessment of tympanometry type B or C

Table 3. Comparison of demographic and clinical characteristics across cross-validation folds (ear level)

	**Fold 0**	**Fold 1**	**Fold 2**	**Fold 3**	**Fold 4**	**Fold 5**	**Fold 6**	**Fold 7**	**Fold 8**	**Fold 9**
Grade category – N (%)										
ECE	18 (3.7%)	25 (5.2%)	32 (6.7%)	28 (5.8%)	38 (7.9%)	34 (7.1%)	30 (6.2%)	28 (5.8%)	22 (4.6%)	24 (5.0%)
K-5	250 (51.9%)	229 (47.5%)	243 (50.7%)	250 (52.0%)	251 (52.2%)	241 (50.1%)	236 (49.1%)	251 (52.2%)	254 (52.8%)	246 (51.1%)
6-8	102 (21.2%)	118 (24.5%)	120 (25.1%)	99 (20.6%)	107 (22.2%)	103 (21.4%)	122 (25.4%)	126 (26.2%)	111 (23.1%)	94 (19.5%)
9-12	112 (23.2%)	110 (22.8%)	84 (17.5%)	104 (21.6%)	85 (17.7%)	103 (21.4%)	93 (19.3%)	76 (15.8%)	94 (19.5%)	117 (24.3%)
Age category – N (%)										
3-6	80 (16.6%)	68 (14.1%)	76 (15.9%)	60 (12.5%)	91 (18.9%)	98 (20.4%)	104 (21.6%)	67 (13.9%)	81 (16.8%)	88 (18.3%)
7-9	126 (26.1%)	128 (26.6%)	129 (26.9%)	141 (29.3%)	122 (25.4%)	133 (27.7%)	117 (24.3%)	130 (27.0%)	138 (28.7%)	132 (27.4%)
10-12	135 (28.0%)	110 (22.8%)	149 (31.1%)	132 (27.4%)	138 (28.7%)	96 (20.0%)	112 (23.3%)	145 (30.1%)	115 (23.9%)	111 (23.1%)
13-15	54 (11.2%)	122 (25.3%)	76 (15.9%)	82 (17.0%)	71 (14.8%)	97 (20.2%)	90 (18.7%)	89 (18.5%)	91 (18.9%)	78 (16.2%)
16-18+	87 (18.0%)	52 (10.8%)	49 (10.2%)	66 (13.7%)	57 (11.9%)	57 (11.9%)	58 (12.1%)	48 (10.0%)	56 (11.6%)	72 (15.0%)
Female, N(%)	240 (49.8%)	210 (43.6%)	174 (36.3%)	238 (49.5%)	205 (42.6%)	253 (52.6%)	253 (52.6%)	204 (42.4%)	208 (43.2%)	246 (51.1%)
Alaska Native/American Indian, N(%)	464 (96.3%)	464 (96.3%)	459 (95.8%)	444 (92.3%)	463 (96.3%)	468 (97.3%)	468 (97.3%)	463 (96.3%)	473 (98.3%)	465 (96.7%)
Highest education of any caregiver, N (%)										
<12 grade	35 (7.3%)	30 (6.2%)	10 (2.1%)	28 (5.8%)	28 (5.8%)	28 (5.8%)	23 (4.8%)	19 (4.0%)	45 (9.4%)	32 (6.7%)
HS Diploma or GED	311 (64.5%)	307 (63.7%)	334 (69.7%)	333 (69.2%)	305 (63.4%)	282 (58.6%)	326 (67.8%)	264 (54.9%)	296 (61.5%)	297 (61.7%)
Some College	88 (18.3%)	101 (21.0%)	87 (18.2%)	57 (11.9%)	109 (22.7%)	108 (22.5%)	79 (16.4%)	132 (27.4%)	96 (20.0%)	74 (15.4%)
College Degree	38 (7.9%)	40 (8.3%)	36 (7.5%)	44 (9.1%)	31 (6.4%)	50 (10.4%)	49 (10.2%)	47 (9.8%)	44 (9.1%)	70 (14.6%)
Tympanometry-type										
A	425 (88.2%)	431 (89.4%)	427 (89.1%)	454 (94.4%)	437 (90.9%)	439 (91.3%)	414 (86.1%)	434 (90.2%)	432 (89.8%)	437 (90.9%)
B	36 (7.5%)	39 (8.1%)	35 (7.3%)	19 (4.0%)	30 (6.2%)	33 (6.9%)	48 (10.0%)	34 (7.1%)	41 (8.5%)	30 (6.2%)
C	21 (4.4%)	12 (2.5%)	17 (3.5%)	8 (1.7%)	14 (2.9%)	9 (1.9%)	19 (4.0%)	13 (2.7%)	8 (1.7%)	14 (2.9%)
Middle ear disease, N(%)	74 (15.4%)	57 (11.8%)	66 (13.8%)	36 (7.5%)	55 (11.4%)	47 (9.8%)	75 (15.6%)	58 (12.1%)	55 (11.4%)	53 (11.0%)

Note: there were 6 missing values for child age across the 10 folds.
Note: there were 2 missing values for child gender in fold 3, none anywhere else.
Note: there were 97 missing values for max parental education across the 10 folds.
Note: there were 4 missing values for middle ear disease across the 10 folds.

Table 4. Confusion matrix for 3-level classification using layman tracings

	Layman Tracing											
	**Built-in Software[1]**				**Simple Decision Tree[1]**				**Hybrid Deep Learning[2]**			
Ground Truth	A	B	C	Total	A	B	C	Total	A	B	C	Total
A	4096	95	139	4330	4142	175	13	4330	3998	283	49	4330
B	70	260	15	345	156	180	9	345	13	315	17	345
C	30	24	81	135	51	7	77	135	10	28	97	135
Total	4196	379	235	4810	4349	362	99	4810	4021	626	163	4810
Sensitivity	94.6	75.4	60.0		95.7	52.2	57.0		92.3	91.3	71.9	
Specificity	79.2	97.3	96.7		56.9	95.9	99.5		95.2	93.0	98.6	

[1]Computed as simple cross-tabulation across all observations
[2]Computed as sum of confusion matrices from 10 test sets

Table 5. Confusion matrix for 3-level classification using audiologist tracings

	Audiologist Tracing											
	**Built-in Software[1]**				**Simple Decision Tree[1]**				**Hybrid Deep Learning[2]**			
Ground Truth	A	B	C	Total	A	B	C	Total	A	B	C	Total
A	4158	21	151	4330	4158	165	7	4330	4232	62	36	4330
B	68	262	15	345	148	192	5	345	18	318	9	345
C	39	17	79	135	28	8	99	135	5	16	114	135
Total	4265	300	245	4810	4334	365	111	4810	4255	396	159	4810
Sensitivity	96.0	75.9	58.5		96.0	55.7	73.3		97.7	92.2	84.4	
Specificity	77.7	99.1	96.4		63.3	96.1	99.7		95.2	98.3	99.0	

[1]Computed as simple cross-tabulation across all observations
[2]Computed as sum of confusion matrices from 10 test sets

Table 6. Confusion matrix for pass/refer classification using layman tracings

	Layman Tracing								
	**Built-in Software[1]**			**Simple Decision Tree[1]**			**Hybrid Deep Learning[2]**		
Ground Truth	Pass	Refer	Total	Pass	Refer	Total	Pass	Refer	Total
Pass	4096	234	4330	4142	188	4330	3998	332	4330
Refer	100	380	480	207	273	480	23	457	480
Total	4196	614	4810	4349	461	4810	4021	789	4810

[1]Computed as simple cross-tabulation across all observations
[2]Computed as sum of confusion matrices from 10 test sets

Table 7. Confusion matrix for pass/refer classification using audiologist tracings

	Audiologist								
	**Built-in Software[1]**			**Simple Decision Tree[1]**			**Hybrid Deep Learning[2]**		
Ground Truth	Pass	Refer	Total	Pass	Refer	Total	Pass	Refer	Total
Pass	4158	172	4330	4158	172	4330	4232	98	4330
Refer	107	373	480	176	304	480	23	457	480
Total	4265	545	4810	4334	476	4810	4255	555	4810

[1]Computed as simple cross-tabulation across all observations
[2]Computed as sum of confusion matrices from 10 test sets

Table 8. Comparison of sociodemographic and clinical characteristics between child/ear/years where tracings differed diagnostically between audiologist and layman (i.e. the 3.01%)

**Reason for discrepancy**		
Audiologist Error	12 (7.7%)	
Layman Error	69 (44.2%)	
Layman-Probe/Wall	44 (28.2%)	
Machine Error	1 (0.6%)	
Possibly Real Difference	30 (19.2%)	
	**Tracings diagnostically similar (n=4654)**	**Tracings diagnostically different (n=156)**
Age category – N (%)		
3-6	789 (17.0%)	24 (15.4%)
7-9	1247 (26.8%)	49 (31.4%)
10-12	1204 (25.9%)	39 (25.0%)
13-15	824 (17.7%)	26 (16.7%)
16-18+	584 (12.5%)	18 (11.5%)
Grade category – N (%)		
ECE	272 (5.8%)	7 (4.5%)
K-5	2366 (50.8%)	85 (54.5%)
6-8	1066 (22.9%)	36 (23.1%)
9-12	950 (20.4%)	28 (17.9%)
Female, N(%)	2172 (46.7%)	59 (37.8%)
Tympanometry type		
A	4228 (90.8%)	102 (65.4%)
B	324 (7.0%)	21 (13.5%)
C	102 (2.2%)	33 (21.2%)
Middle ear disease, N(%)	514 (11.0%)	62 (39.7%)

Note: there were 6 missing values for child age in the diagnostically similar tracings, none in the diagnostically different tracings.
Note: there were 2 missing values for child gender in the diagnostically similar tracings, none in the diagnostically different tracings.
Note: there were 4 missing values for middle ear disease across all tracings.

Figure 1. ROC curves of Hybrid Deep Learning Model

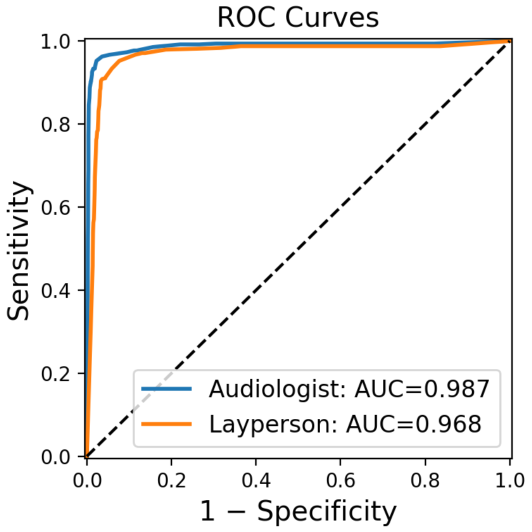

Supplement: Supplementary file 1 [file aud-44-1262-s001.pdf]
